# Practical Demonstration of the Human Skeleton

**Published:** 1837-01

**Authors:** 


Art. IX.—*A Practical Demonstration of the Human Skeleton.* By George Elkington, m.r.c.s., Demonstrator of Anatomy in the Royal School of Medicine and Surgery, Birmingham.—*London*, 1836. 12mo. pp. 240.

This little work is very creditable to its author, yet we cannot think that it was called for by any lack of such helps in the schools. As stated in the preface, it certainly contains a minuter description of the bones than any of the Manuals in common use, and it is inferior to none in accuracy. It wants, no doubt, all the charm of raciness which attaches to some of the older works on the same subject, particularly that of Monro: it wants equally the elegance, perspicuity, and conciseness of the recent production of Dr. Quain; but, to such students as are blest with an industry which tires not, (and we may say that they cannot have a too minute knowledge of osteology,) its copiousness must be both delightful and precious. We remark one defect in it, which, although apparently slight, is not really so; we mean the total want of Latin synonyms. Although it is far from desirable to burden the memory of the student with unnecessary terms, still, to say nothing of the advantage to him in a literary point of view, it is essential that he should be familiar with names which are in such general use, and which he is constantly liable to meet with in his perusal of anatomical and surgical works, particularly of the older and of foreign authors.

